# Supplementary figures and images for: PAIRNet: Predicting PIWI cleavage specificity via position-aware RNA interaction modeling
Source: PLoS Comput Biol. 2026 Feb 19;22(2):e1013936. doi: 10.1371/journal.pcbi.1013936 (PMC12919788; doi:10.1371/journal.pcbi.1013936)

**A**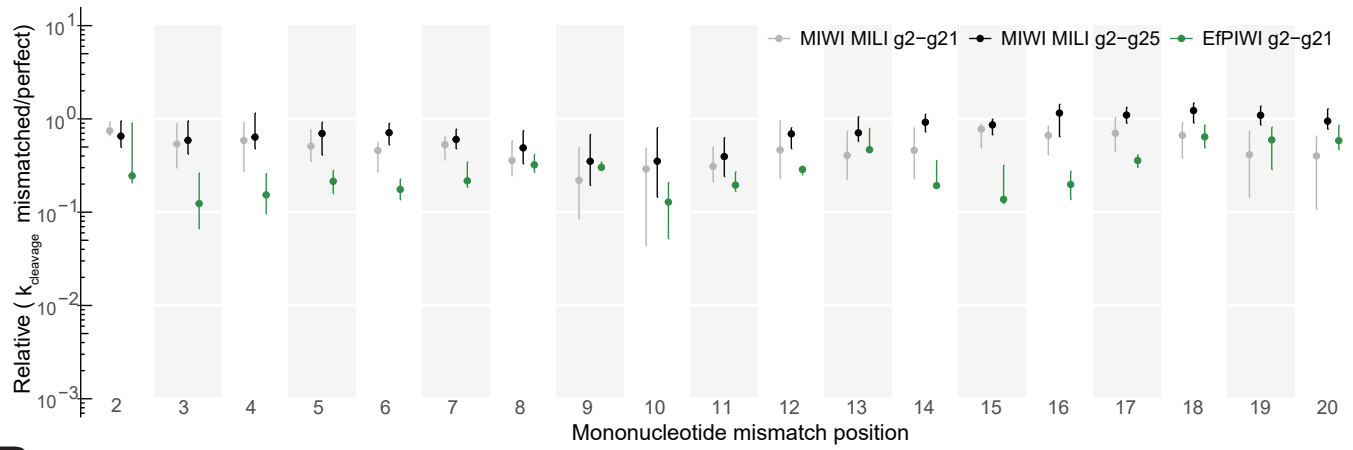**B**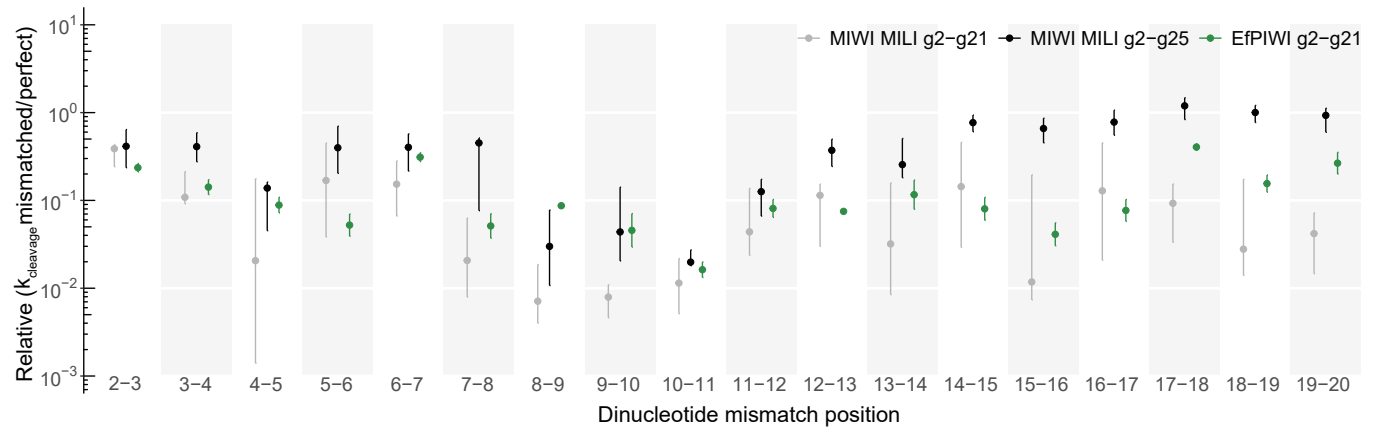**C**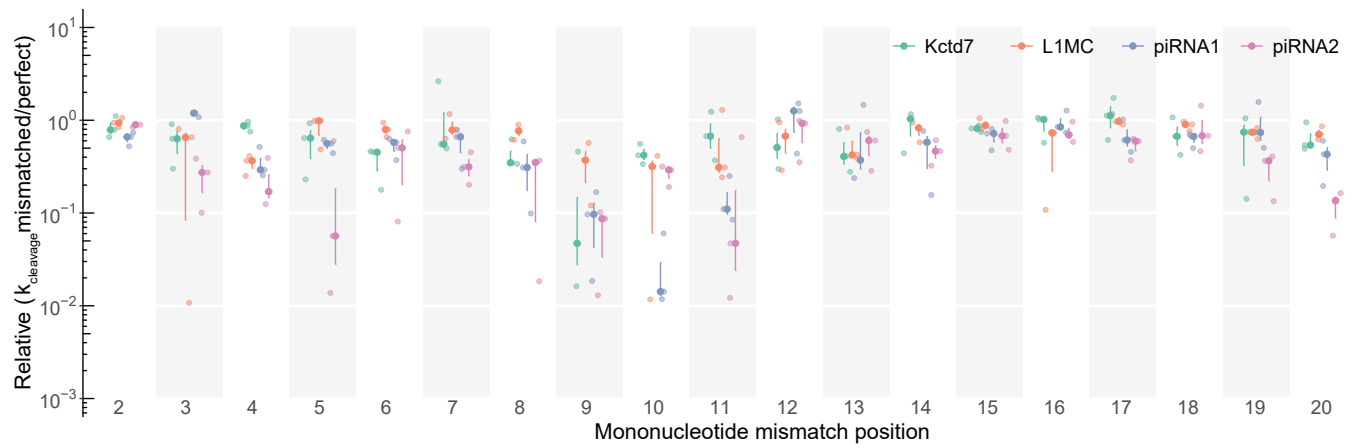**D**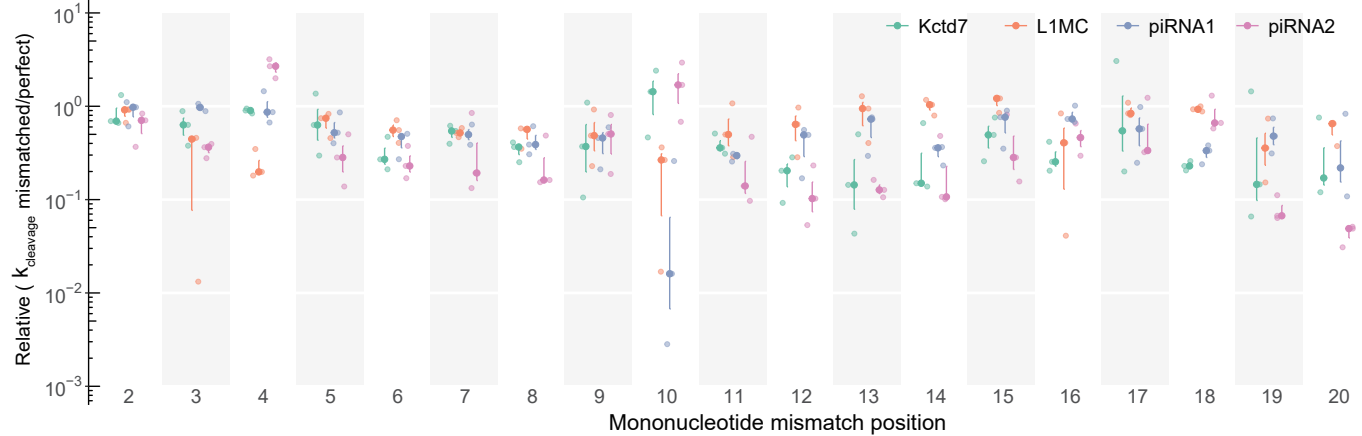

Supplement: S1 Fig — Pre-steady-state cleavage rates for targets containing (A) single or (B) two consecutive mismatches between guide positions g2 and g20. Data includes MILI, MIWI, and EfPiwi with contiguous pairing extending to g21 or g25. Expanded k distributions for single-mismatch targets across four distinct guide RNAs loaded into (C) MILI and (D) MIWI. Each dot represents a specific mismatch geometry within the g2–g20 region (g2–g21 pairing). Median and interquartile range (IQR) are shown for all panels. (PDF) [file pcbi.1013936.s001.pdf]

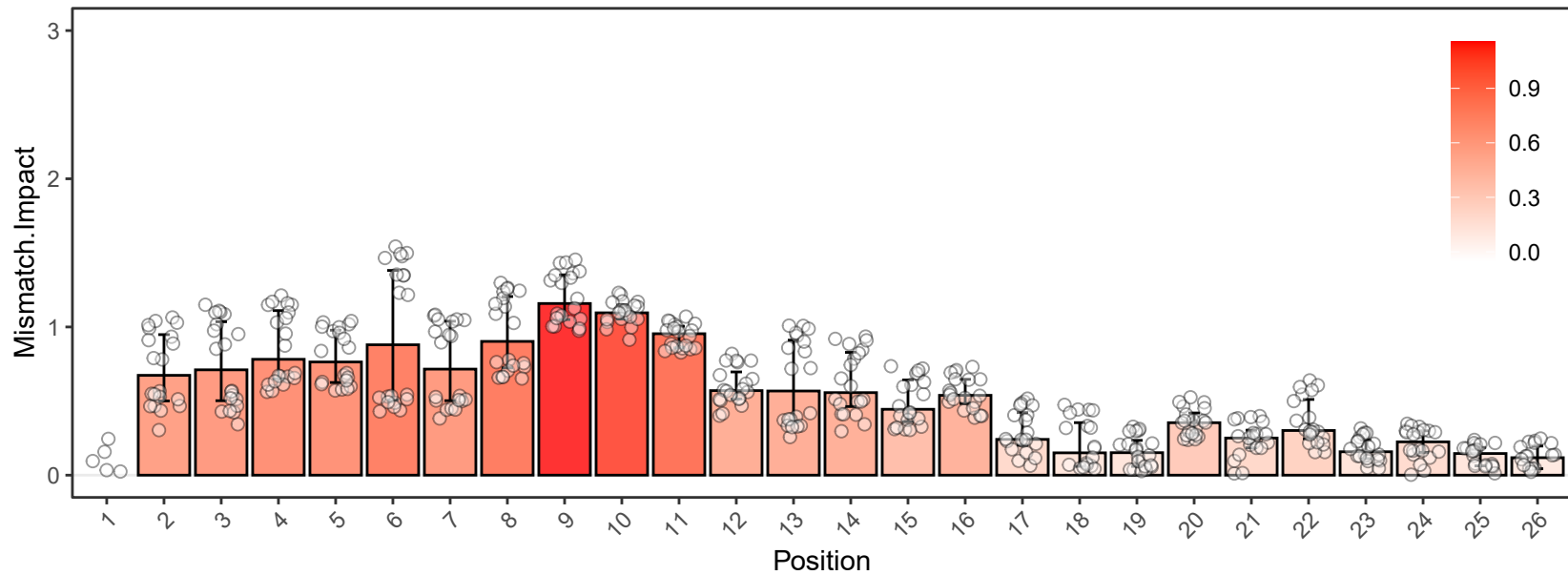

Supplement: S2 Fig — Median impact scores derived from the PAIRNet Mixed Model (integrating both MIWI and MILI datasets). The mixed model retains the feature importance profile centered on the catalytic core (g10–g11), though with higher variance due to subtle kinetic differences between PIWI homologs. (PDF) [file pcbi.1013936.s002.pdf]

**A**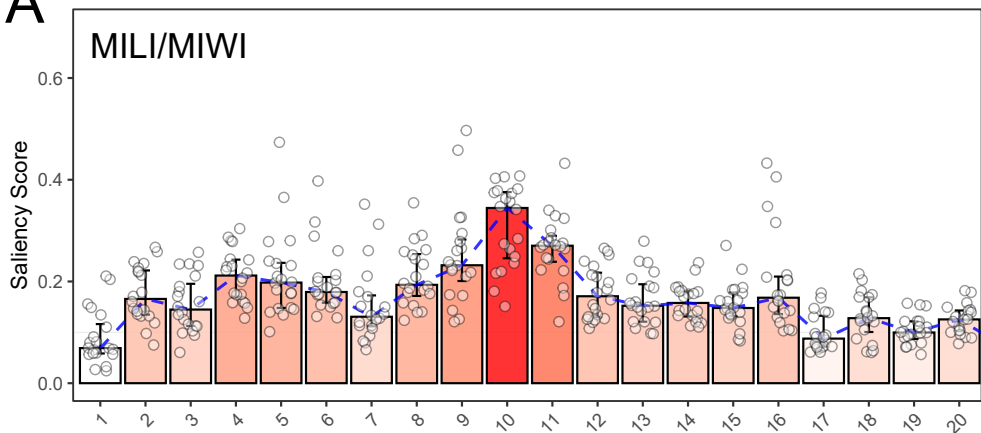**B**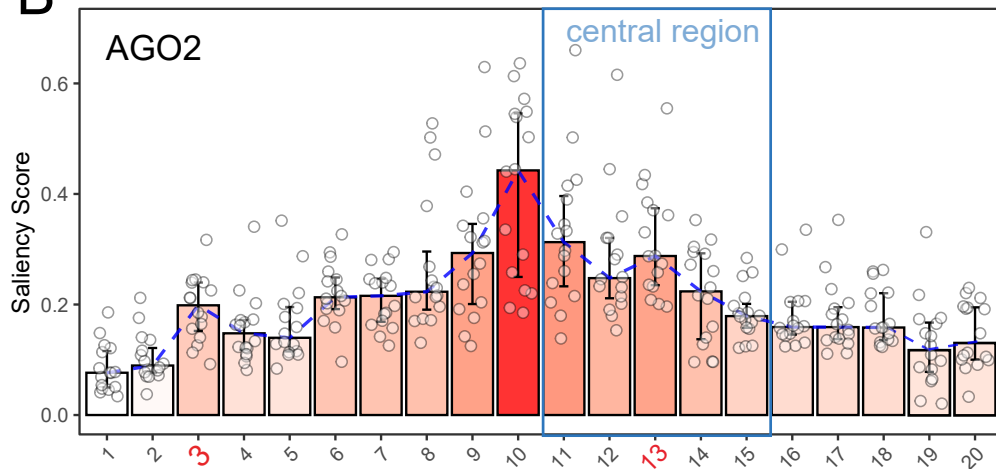

Supplement: S3 Fig — Saliency score are calculated for MILI/MIWI (A) and AGO2 (B), Position 3 and 13 are important positions mentioned by Becker et al. [15]. (PDF) [file pcbi.1013936.s003.pdf]
