# Supplementary material for: PAIRNet: Predicting PIWI cleavage specificity via position-aware RNA interaction modeling
Source: PLoS Comput Biol. 2026 Feb 19;22(2):e1013936. doi: 10.1371/journal.pcbi.1013936 (PMC12919788; doi:10.1371/journal.pcbi.1013936)
Supplement: Attachment — Submitted filename: Point_by_Point_response.pdf [file pcbi.1013936.s007.pdf]

Dear Editors and Reviewers,

We are very grateful for the constructive comments and questions. These suggestions help us greatly improve the scientific rigor of our study. We have fully accepted your advice and have made extensive corrections in the manuscript, including new validations and mechanistic comparisons. Detailed responses to each specific comment are provided below.

We organize our rebuttal as follows. To answer each question, we first outline the scientific logic of our approach, clarifying the rationale behind our answer. We then provide a detailed answer to the reviewer's question, supported by quantitative evidence derived from new experiments, additional analyses, or citations of relevant literature. Finally, we summarize the specific revisions made to the manuscript, clearly indicating where the text has been modified, inserted, or reorganized. To facilitate review, the corresponding changes in the manuscript are explicitly quoted at the end of each response, highlighting the exact location and content of the revision. To facilitate the review process, all revisions in the "Marked-up Copy" of the manuscript have been highlighted in red text.

Reviewer 1#:

The manuscript by Zeng et al describes a new deep learning framework that captures targeting rules by PIWI proteins. This is an important unaddressed question and the authors were able to create a model that mostly recapitulates the biochemical experiments. However, several key questions remain unanswered.

Q1: Can authors' model explain why there is difference between different guide piRNAs?

Response:

We thank the reviewer for raising this fundamental question. Understanding the intrinsic determinants of guide RNA efficacy, why one piRNA is inherently a more potent silencer than another even under the same pairing scenario like fully match, remains one of the most significant and challenging open problems in the field of RNA interference. Deciphering the specific sequence or structural features that govern guide-specific potency is critical not only for a deep mechanistic understanding of genome defense but also for the rational design of effective RNA therapeutics. Wang & Bartel reported that quantitative profiling of human AGO2 loaded with diverse miRNA sequences reveals that cleavage efficiency for perfectly paired targets can

span a range of greater than 250 fold [1]. This means that one guide RNA might direct the cleavage of a target in seconds, while another guide, equally well-paired, might require minutes to hours to achieve the same chemical event. Even with perfect target complementarity, Argonaute cleavage efficiency is primarily dictated by specific guide RNA sequence features, most notably a purine at position 10, A/U residues at positions 7 and 17, and shorter guide length, which collectively facilitate the rate-limiting structural transition of the RISC complex into its catalytically active "slicing-competent" conformation [1].

Our model successfully captures latent differences between guide RNAs to some extent, as evidenced by our experiments by explicitly encoding the guide sequence (G) alongside the interaction matrix (Figure 2A). PAIRNet effectively learns intrinsic features of the guide (such as sequence composition biases) that influence cleavage efficiency. This is empirically supported by our "leave-one-guide-out" validation experiment. When we trained PAIRNet on three guide datasets and tested it on the fourth unseen guide, we observed variations in predictive performance (e.g., lower PCC for piRNA#2 compared to L1MC, Figure 4). This variation indicates that our model is not treating all guides as identical; rather, it is sensitive to the specific sequence features of each guide. If the model were unable to reflect the differences between guides, the performance drop on the unseen guides would likely be uniform or random, rather than guide-specific.

However, a complete mechanistic explanation remains challenging due to data scarcity. Current high-throughput datasets, including those utilized in this study, cover only four distinct guide sequences. While this volume of data is sufficient to model guide-target interactions, it is insufficient to fully generalize the guide-intrinsic rules (efficacy) across the vast piRNA repertoire. Deep learning models rely on high-diversity training data. With only four distinct "labels" for guide identity, the model faces a significant obstacle in disentangling global guide-efficacy rules from guide-specific noise.

To overcome these barriers and achieve a fully explanatory model, we have outlined a strategy for future work: 1) Expanding Data Diversity: To address the data shortage, our laboratory is currently generating new Cleave-N'-Seq libraries using a standardized protocol to systematically test a broader array of guide sequences. This will provide the necessary training data to move beyond the current limited set and capture subtle sequence determinants that decouple loading from slicing; 2) We hypothesize that guide-specific efficacy differences are driven by the energetic cost of transitioning to the slicing-competent conformation, governed by steric constraints involving not only the anchoring domains but critically the N-domain and the catalytic channel. Since PAIRNet currently models the 2D RNA-RNA duplex, it cannot capture 3D determinants such as the propagation of the distal seed through the central cleft (nucleotides 6–7) or the steric release of the 3' end from the PAZ domain at position 17 [1, 2]. Future iterations of our framework will incorporate 3D geometric deep learning, trained on high-resolution exited co-crystal structures, to explicitly model the dynamic protein-RNA interface and pinpoint the atomic-level causes of

differential cleavage efficiency.

Corresponding Manuscript Revision colored red words inserted (Results Line # 567**)**

PAIRNet's strengths lie in its biological interpretability, high accuracy, and scalability. By quantifying position-specific mismatch penalties and insertion impacts, it provides actionable rules for designing piRNA guides with enhanced specificity. However, there are still limitations. The model is trained on in vitro CNS-seq data, which may not fully recapitulate in vivo complexity (e.g., chromatin effects, co-factors like GTSF1). Furthermore, PAIRNet is trained on mammalian PIWI data, which reflects a 'relaxed' targeting mechanism. Consequently, the current model may not fully generalize to other systems, such as the silkworm Siwi-RE, where a 'locked' conformation requires extensive 3' pairing to structurally activate the complex. Additionally, the current dataset's limited guide sequence diversity restricts the model's ability to fully generalize intrinsic guide efficacy rules. Future work should expand to in vivo cleavage assays, additional PIWI homologs (e.g., HIWI, HILI), and other species-specific data. Integrating 3D structural data (e.g., PIWI-RNA cocrystal structures) may further refine feature representations. Finally, extending PAIRNet to predict off-target effects or synergize with CRISPR-based systems may unlock new avenues for RNA-guided genome engineering. While PAIRNet effectively flags the structural disruption caused by insertions and deletions, our current encoding does not distinguish the specific nucleotide identity of the inserted base. Future iterations of the framework could incorporate the thermodynamic contributions of specific indel sequences (e.g., differentiating stabilizing vs. destabilizing bulges) to further refine prediction accuracy.

Q2: Cleave-n-Seq data also exists for Ago2, and authors could use their approach to create a second model for AGO proteins and compare the two.

Response:

We sincerely thank the reviewer for this constructive suggestion. We agree that modeling AGO2, which is the best-characterized member of the Argonaute family, provides an essential baseline for validating PAIRNet's ability to capture distinct, lineage-specific targeting mechanisms. Unlike PIWI proteins, which is shown to exhibit "relaxed" targeting rules and a reliance on auxiliary factors [3], AGO2 operates under significantly stricter geometric constraints, particularly regarding the rigidity of seed pairing and the central base pairing checkpoint. Extending PAIRNet to learn these distinct AGO2-specific rules allows us to rigorously stress-test our framework's generalizability, proving that its interaction-centric architecture can decipher the unique biophysical 'fingerprints' of different Argonaute subclades rather than simply memorizing a single targeting paradigm.

To address this, we curated and processed AGO2 CNS-seq data from two

independent studies to build a dedicated PAIRNet-AGO2 model. Because these datasets employed different library construction strategies, we performed a rigorous re-analysis from raw data to ensure validity. The L1MC-AGO2 dataset from *Gainetdinov et al.* (2023) [3] was generated using a library of short, uniform 30-nucleotide target RNAs designed to isolate specific mismatch effects in a minimal context. Conversely, the let-7a and miR-21 AGO2 datasets from *Becker et al.* (2019) [2] employed a complex library design, utilizing variable-length constructs that incorporated diverse flanking sequences and specific randomized contexts to query secondary structure effects. These distinct approaches to substrate design, comparing short, fixed-length targets against extended transcripts with variable flanking regions, introduce significant experimental heterogeneity regarding target accessibility and folding, which precludes the integration of these datasets into a model training for a robust AGO2 model. Therefore, before modeling, we performed a rigorous re-analysis from raw data to ensure validity.

We aggregated AGO2 cleavage data for three distinct guides: let-7a and miR-21 from *Becker et al.* (2019)[2], and L1MC from *Gainetdinov et al*. (2023)[3].

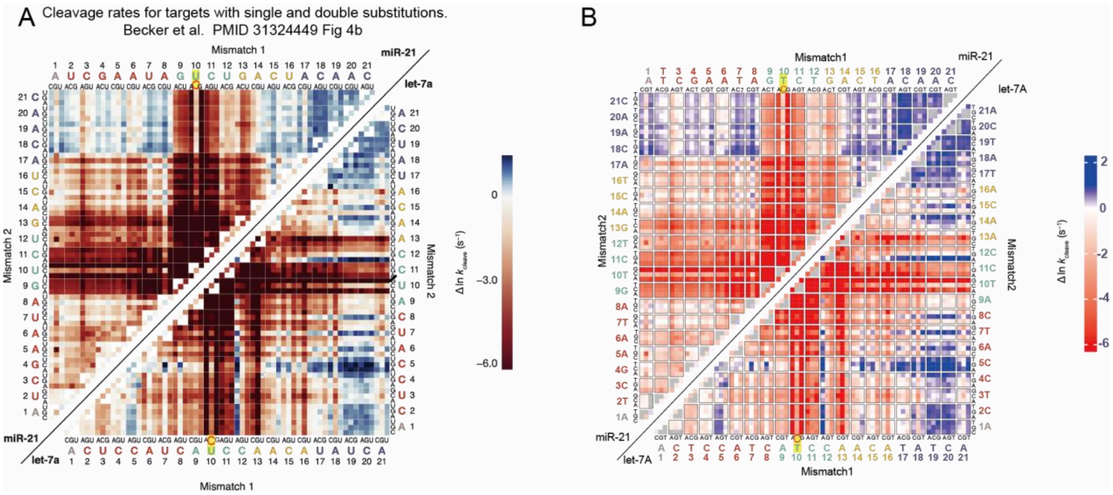

Figure R1. (A) is the raw Figure 4b in *Becker et al*. [2]. It visualizes the mutation cleavage rate for targets with single and double substitutions, cleavage rates for miR-21 (upper left) and let-7a (lower right) targets with single and double substitutions. Deep red represents targets for which no detectable cleavage was observed. Targets colored in blue were cleaved faster than the fully complementary target. (B) is our reproduction result, by using supplementary file from *Becker et al*. [2] to repeat the analysis in (A). Highlighted position indicates the observation that T-to-C substitutions at g10 result in higher cleavage rates (*k*) compared to T-to-A or T-to-G substitutions.

Processing Becker et al. (let-7a/miR-21) [2]: We extracted cleavage rates for targets containing single and double substitutions. To validate our understanding of this complex library, we attempted to replicate Figure 4B from the original publication. Our replication in Response Figure R1(B) matched the original report in Figure R1(A) almost exactly. We successfully captured the critical impact of substitutions at positions 9, 10, and 11. Furthermore, our re-analysis reproduced subtle

variations reported in the original study, such as the observation that T-to-C substitutions at g10 result in higher cleavage rates (*k*) compared to T-to-A or T-to-G substitutions (See highlighted positions in Figure R1). This confirmed the accuracy of our data intake for these two guides.

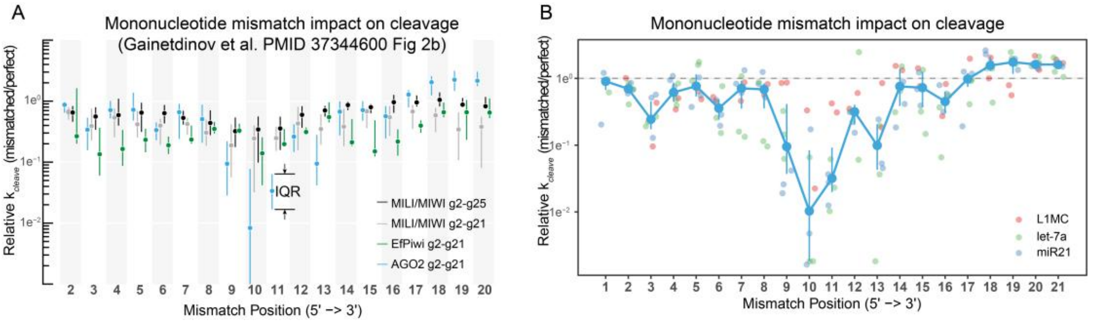

Figure R2. (A) Change in pre-steady-state (*k*) cleavage rate for one or two consecutive mismatches between g2 and g20 for contiguous g2–g21 or g2–g25 pairing for MILI, MIWI, E*f*Piwi and mouse AGO2. Median and IQR are shown. This is raw figure of Figure 2b in Gainetinov et al. [3]. (B) the same plot we try to repeat from raw data.

Processing Gainetdinov et al. (L1MC) [3]: Since the per-target source data for the AGO2-L1MC experiment was not provided in the original paper (unlike the PIWI data), we downloaded the raw sequencing reads from the SRA database. We processed 36 libraries (3 biological replicates × 12 time points: 0-short, 1, 2, 4, 8, 0-long, 20, 40, 60, 240, 480, and 960 minutes). The SRA accessions included SRR25009230, SRR25009274, SRR25009277-SRR25009281, SRR25009284, SRR25009288-SRR25009297, SRR25009360-SRR25009363, SRR25009365, SRR25009371, SRR25009376-SRR25009478, SRR25009588-SRR25009590, and SRR25009592-SRR25009597. Data analysis pipeline is coherent to https://github.com/ildargv/Gainetdinov_et_al_2023. We attempted to replicate Figure 2b from the original paper [3]. Our analysis in Figure R2(B) successfully reproduced the original trends as given in Figure R2(A), specifically highlighting the drastic reduction in cleavage rate caused by single mismatches at the 10th position, relative to the changes at the 9th and 11th positions.

PAIRNet AGO2 Model Training: Given the limited dataset of only three guide RNAs for AGO2 compared to the PIWI dataset, we modified our training strategy. Rather than the “leave-one-out” cross-validation used for the PIWI model, we pooled the let-7a, miR-21, and L1MC datasets into a unified training dataset. Using optimized hyperparameters (α=0.8 and a learning rate of 0.0001), the resulting PAIRNet-AGO2 model demonstrated remarkable predictive power, achieving a Pearson Correlation Coefficient (PCC) of 0.769±0.0276.

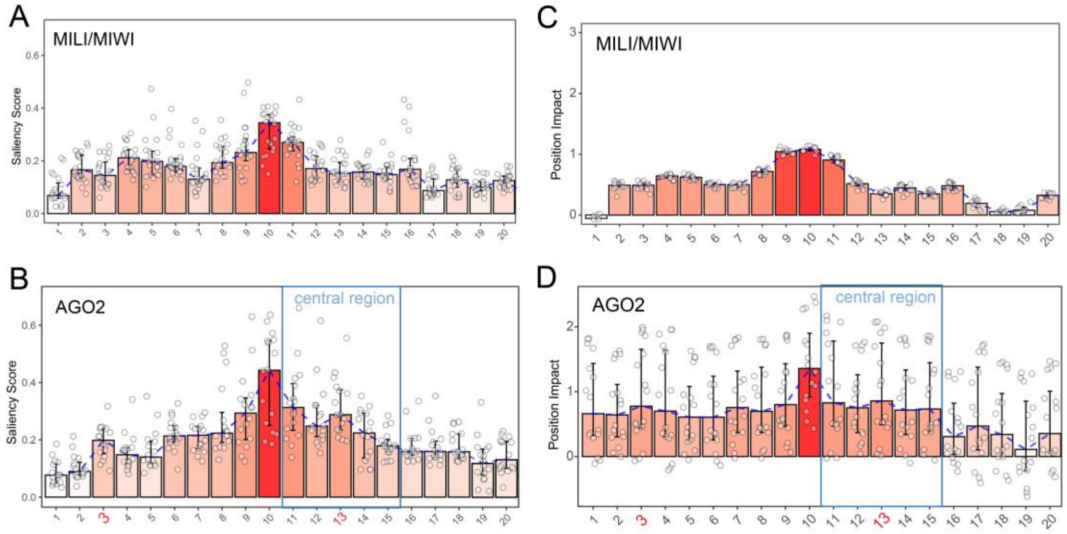

Figure R3. Position importance analyzed for AGO2 and MILI/MIWI by saliency scoring and counterfactual analysis. Saliency scores are calculated for MILI/MIWI (A) and AGO2 (B), and simultaneously we did counterfactual analysis for MILI/MIWI (C) and AGO2 (D). Position 3 and 13 are important positions mentioned by *Becker et al* [2].

Comparative Interpretability: To rigorously analyze the mechanistic divergence between the two Argonaute families, we subjected the new PAIRNet-AGO2 model to the same Saliency Scoring and Counterfactual Analysis used for our original PIWI model (Figure R3). The comparative results reveal a biological distinction that quantitatively mirrors the structural constraints reported in the literature. Specifically, the AGO2 model exhibits a contrast in feature importance, characterized by a sharp, high-magnitude peak centered on the catalytic core (g10) and extending significantly into the central region (g11–g15) (Figure R3). This aligns with biochemical data showing that while AGO2 can tolerate certain backbone distortions, it is highly sensitive to base mismatches at the cleavage site [2]. *Becker et al.* explicitly identify position 13 as a critical determinant for AGO2 specificity, stating that discriminating between targets "was most effective when the mismatch was at position 3 or position 13 [2]" because mismatches here cause a "decrease in cleavage rate". The model's heavy emphasis on this region correctly reflects the biological reality established by *Gainetdinov et al.*, who noted that single mismatches near the AGO2 scissile phosphate abolish cleavage activity by 10- to 200-fold.

Conversely, the PIWI model displays a distributed importance profile with lower relative penalties at the catalytic center. This is consistent with the "relaxed targeting" described by *Gainetdinov et al*., who observed that PIWI proteins, aided by the auxiliary factor GTSF1, retain cleavage activity even when positions flanking the scissile phosphate are mismatched (resulting in a median ~4-fold reduction)[3]. The PAIRNet-AGO2 model's sensitivity to the g11–g15 window indicates that the network captures the stricter pairing requirements of the AGO2 central cleft relative to the mechanism of PIWI proteins.

Furthermore, both models assign lower importance to the 3' end of the guide

(g16–g21). This aligns with biochemical evidence that strong 3’ pairing is not required for cleavage in either family and can potentially reduce reaction turnover by slowing product release. Although the AGO2 analysis showed greater variance, likely due to differences in target lengths within the training data, the results clearly distinguish the strict seed and central pairing requirements of AGO2 from the more flexible targeting rules of PIWI.

We observed a difference between the results by the two interpretability metrics for AGO2. While the Saliency Score in Figure R3(B) indicates a specific importance peak at the central core, the Counterfactual Analysis in Figure R3(D) shows high importance across the seed-to-central window (g3-g15). This uniformity likely reflects the sensitivity of the counterfactual calculation to effective zero-activity states. Because AGO2 cleavage is sensitive to single mismatches in both the seed (which affect binding) and the central region (which affect catalysis), perturbations in either region can reduce predicted activity to near zero. When mismatches in different regions result in similar low-activity predictions, the calculated penalties become indistinguishable. Therefore, the plateau indicates that seed and central mismatches have comparable impacts on the final cleavage prediction for AGO2, in contrast to the mismatch-tolerant profile observed in PIWI proteins.

Corresponding Manuscript Revision red colored words inserted (Results Line # 498**):**

PAIRNet generalizes to AGO2 and captures distinct lineage-specific targeting rules. To evaluate PAIRNet’s ability to generalize beyond PIWI proteins, we retrained the framework on mouse AGO2 Cleave-N’-Seq data. Unlike PIWI proteins, which utilize a ‘relaxed’ targeting mechanism, AGO2 enforces stricter geometric constraints, particularly in the central cleft. The AGO2-specific model achieved a PCC of 0.769±0.0276 . Critically, comparative interpretability analysis revealed a sharp divergence in feature importance: while the PIWI model displays distributed importance compatible with mismatch tolerance, the AGO2 model exhibits a high-magnitude saliency peak centered on the catalytic core and central region (g10-g15) (S2 Fig). This aligns with structural evidence that AGO2 is highly sensitive to mismatches near the scissile phosphate, confirming that PAIRNet captures the distinct biophysical fingerprints of different Argonaute families rather than memorizing a single rule set.

Q3: Ideally, authors model should be accessible not only as a raw code, but also a convenient tool for prediction of targeting rules for a specific piRNA sequence.

Response:

We appreciate the reviewer’s suggestion to improve accessibility of our method. Our model learns universal biophysical rules during training to predict cleavage efficacy for specific sequences during inference. It does not generate unique rules for

individual piRNAs, but rather applies the learned understanding of steric and energetic constraints to predict how well a specific piRNA will target a given substrate.

In response, we have developed a web-based server for PAIRNet (available at https://scgerm-atlas.sjtu.edu.cn/pairnet/pairnet.html). The PAIRNet Cleavage Predictor serves as a deep-learning tool to quantify the silencing efficiency of piRNA-target interactions, moving beyond classical thermodynamic assumptions by utilizing an ensemble of neural networks trained on high-throughput cleavage data. Its primary advantage lies in its ability to contextualize raw predictions, automatically benchmarking the user's specific target variant against a computer-generated perfect match to provide a biologically interpretable relative cleavage score accompanied by a confidence interval that reflects the consensus among the underlying models. To operate the tool effectively, researchers must provide precise 26-nucleotide sequences, specifically a 5'-to-3' guide and a 3'-to-5' target, along with a detailed pairing mode string, ensuring the input aligns with the model's strict training parameters. While highly accurate for characterizing substitution mutations and mismatch penalties, the tool is currently limited to these specific length constraints and mismatch scenarios, as the non-linear structural complexities introduced by insertions, deletions, or variable-length sequences are not yet supported by this specific inference framework. The detailed tutorial is shown on the website.

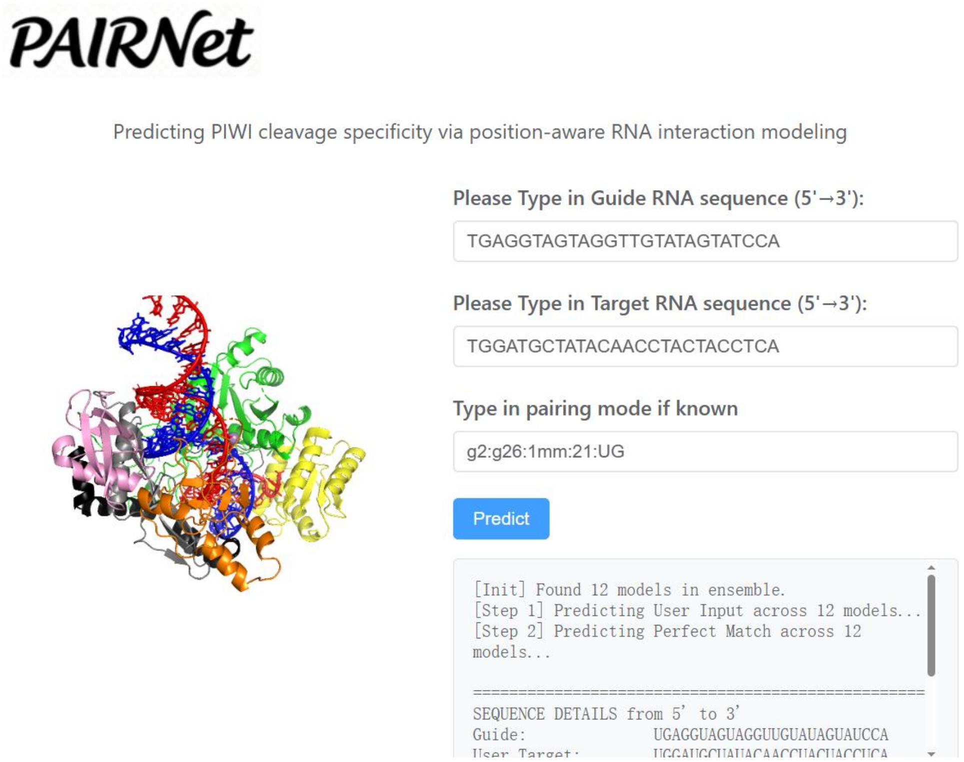

Figure R4. A screen capture of PAIRNet web-based prediction tool for a given piRNA and a target. The input follows the basic logic that PAIRNet prediction of relative *k* value based on guide RNA, target and pairing mode, which is artificially designed by the biologists.

For demonstration, you can try the following three groups of predictions, copy and paste the guide RNA, target RNA and pairing mode into the textbox, and press PREDICT button, then you get the relative *k* ratio, from this demonstration, you get a sense that g10 is much more important than g3 and g20.

Group1, Mutation at g10 (central region)

Guide RNA: TGAGGTAGTAGGTTGTATAGTATCCA

Target RNA: TGGATACTATACAACCGACTACCTCA

Pairing Mode: g2:g26:1mm:10:AG

====================================

ENSEMBLE RESULTS (Median of 10 models)

RELATIVE K RATIO: 0.1641

=====================================

Mutation at g3 (Seed Region)

Guide RNA: TGAGGTAGTAGGTTGTATAGTATCCA

Target RNA: TGGATACTATACAACCTACTACCACA

Pairing Mode: g2:g26:1mm:3:AA

====================================

ENSEMBLE RESULTS (Median of 10 models)

RELATIVE K RATIO: 0.5697

Mutation at g20 (3' Region)

Guide RNA: TGAGGTAGTAGGTTGTATAGTATCCA

Target RNA: TGGATGCTATACAACCTACTACCTCA

Pairing Mode: g2:g26:1mm:21:UG

====================================

ENSEMBLE RESULTS (Median of 10 models)

RELATIVE K RATIO: 0.9675

Corresponding Manuscript Revision red colored words inserted (Introduction Line # 106**)**

Together, these features allow PAIRNet to address the limitations of sequence-only models, demonstrating improved predictive accuracy across diverse datasets while offering interpretable insights into PIWI targeting preferences. By connecting computational predictions to structural principle, such as catalytic core rigidity and 3’ flexibility, PAIRNet supports the design of specific piRNA guides and contributes to our understanding of PIWI targeting mechanisms. This study demonstrates the value of modeling interaction geometry in RNA-guided silencing,

offering a computational approach that could inform the development of future RNA-targeting tools. To facilitate community use, we have implemented PAIRNet as a user-friendly web server available at https://scgerm-atlas.sjtu.edu.cn/pairnet/pairnet.html, which automates the feature encoding and cleavage rate prediction for custom guide-target pairs.

Reviewer #2:

This is a study by Shikui Tu and Enzhi Shen's groups. They build a deep-learning model to investigate the piRNA cleavage rule, trained on Cleave-N'-Seq data. This is the first study using deep learning models to investigate piRNA cleavage rule as I know. The model has two main innovations: 1. It encodes interaction information instead of typical one-hot sequence encode. This enables the corporation of mismatches and indels. 2. The model uses a hybrid CNN-transformer architecture, which captures both the local motifs (consecutive pairing) and the global effect of mismatch/indel. The model overall achieves good performance. The authors also interpreted the model and the results are consistent with known knowledges.

Overall, this work is solid and important for the small RNA silencing field. The manuscript is well-written. However, I have several suggestions concerning the feature encoding below:

Major comments:

Q1. In Fig 1D. Mismatches are encoded as "A|T|C|G", how does it performs comparing to simply encode mismatches as "mismatches"? In my opinion, different base pairs has different physical affinity (e.g. C-A are more stable than T-T), encoding mismatches into base pairs would be a better way. The same for insertions and deletions.

Response:

We thank the reviewer for this insightful comment regarding the thermodynamic nuances of RNA base pairing. We completely agree with your assessment that different mismatch types, such as a G-U wobble versus an A-A clash, possess distinct physical affinities and stability profiles that significantly impact cleavage efficiency. We acknowledge that treating mismatches as generic errors would fail to capture these biochemical realities, and we appreciate the opportunity to clarify how our encoding strategy addresses this specific concern.

While Figure 1D illustrates the mismatch encoding as the specific target nucleotide identity (A, T, C, or G), PAIRNet is designed to mathematically reconstruct the specific base-pair identity during processing. Our model simultaneously ingests

the guide nucleotide identity (via the Guide encoding matrix, G) and the target mismatch identity (via the Pairing encoding matrix, $P_i^{'}$), then combine them both into a single feature vector $X_i = [G_i, P_i^{'}]$. Since the neural network processes these two features together, it effectively learns to distinguish specific interactions. For example, if the guide is encoded as Cytosine and the mismatch feature flags Adenine, the model combines these to represent a specific C-A interaction. This distributed encoding allows PAIRNet to capture the physical affinity distinctions you highlighted without requiring a pre-combined one-hot vector for every possible combination.

The necessity of this granular approach is empirically supported by our ablation study results presented in Figure 5 and S6 Table. We compared PAIRNet against a variant termed "SimplePairing", which simplified mismatches to a binary state (Match vs. Mismatch) effectively ignoring the specific nucleotide identity. This simplification significantly degraded performance—for instance, in the MILI Dataset 3, the Pearson Correlation Coefficient dropped from 0.590 to 0.509. This result confirms your intuition that distinguishing the specific physical nature of the mismatch is essential for accurate prediction, and our current encoding strategy successfully preserves this information.

Corresponding Manuscript Revision words inserted (Materials and methods Line # 189**)**

Crucially, we encode the specific identity of the mismatched target nucleotide (A, C, G, or U) rather than utilizing a generic binary mismatch flag. By processing this target identity vector (P') alongside the guide nucleotide identity (G) within the neural network, PAIRNet implicitly learns specific base-pair representations (e.g., distinguishing a destabilizing A-A clash from a structurally tolerant G-U wobble).

Regarding insertions and deletions, our current model prioritizes flagging their presence and location because the resulting structural deformations (such as bulges or gaps) act as the dominant factor disrupting helical geometry. However, we agree with the reviewer's perspective that the specific identity of an inserted or deleted nucleotide contributes to the finer thermodynamic stability of these loops. While our current results demonstrate that flagging the structural disruption provides strong predictive power, we acknowledge that encoding the specific nucleotide identity of indels could further refine prediction accuracy. We appreciate this suggestion and plan to incorporate this granular feature in future iterations of the PAIRNet framework to capture these subtle thermodynamic effects.

Corresponding Manuscript Revision words inserted (Discussion Line # 574**)**

While PAIRNet effectively flags the structural disruption caused by insertions and deletions, our current encoding does not distinguish the specific nucleotide identity of the inserted base. Future iterations of the framework could incorporate the thermodynamic contributions of specific indel sequences (e.g., differentiating stabilizing vs. destabilizing bulges) to further refine prediction accuracy.

Q2. The last paragraph of Introduction seems to be heavily AI edited, and the impact of PAIRNet is exaggerated.

Response:

We thank the reviewer for pointing this out. Upon critical re-evaluation, we recognize that terms in the original draft such as "transcend," "unprecedented accuracy," and "blueprint" were subjective and over-claimed. Furthermore, the claim of "clinical and biotechnological relevance" was exaggerated given the current study's focus on computational validation. We have revised the text to remove those vocabularies, shifting the focus strictly to the demonstrated methodological improvements and the specific biological insights derived from our data.

To address this, we have systematically replaced the exaggerated phrasing with objective, evidence-based terminology.

Corresponding Manuscript Revision words revised (Introduction Line # 106**)**

Together, these features allow PAIRNet to address the limitations of sequence-only models, demonstrating improved predictive accuracy across diverse datasets while offering interpretable insights into PIWI targeting preferences. By connecting computational predictions to structural principle, such as catalytic core rigidity and 3' flexibility, PAIRNet supports the design of specific piRNA guides and contributes to our understanding of PIWI targeting mechanisms. This study demonstrates the value of modeling interaction geometry in RNA-guided silencing, offering a computational approach that could inform the development of future RNA-targeting tools. To facilitate community use, we have implemented PAIRNet as a user-friendly web server available at https://scgerm-atlas.sjtu.edu.cn/pairnet/pairnet.html, which automates the feature encoding and cleavage rate prediction for custom guide-target pairs.

Q3. Fig 3 and 4. I am wondering how PAIRNet compares to other encoding methods + the CNN-transformer hybrid architecture. It is no wonder typical tree-based models (random forest etc.) cannot decode sequences into interactions, which is why they all perform bad in Fig 3.

Response:

We agree with the reviewer's assessment that tree-based models operating on raw sequence data are expected to struggle with resolving thermodynamic interaction rules. To address this, we structured our evaluation as a two-stage process designed to isolate the contribution of the encoding strategy from the contribution of the model architecture.

First, Figure 3 was intended solely to evaluate the input representation. We applied both sequence-based and interaction-centric encodings to the same set of

standard models (including Random Forest and MLP) to assess whether explicit interaction features are necessary for prediction. The results indicated that when provided with interaction-centric features, even standard models achieved positive correlations, whereas sequence-based inputs consistently resulted in poor performance across architectures. This result confirmed that explicitly encoding the guide-target interaction is a prerequisite for effective modeling, regardless of the downstream classifier.

Second, Figure 4 was designed to evaluate the model architecture under standardized conditions. In this comparison, all baseline models (including Random Forest, XGBoost, and KNN) were trained using the interaction-centric encoding established in the first step . This ensured a direct comparison of modeling capabilities rather than input quality. Under these conditions, PAIRNet consistently demonstrated higher Pearson Correlation Coefficients compared to the baseline methods (e.g., in the MILI dataset). This suggests that while interaction-centric encoding improves performance for all models, the hybrid CNN-Transformer architecture provides additional predictive gains by capturing local motifs and global dependencies that standard machine learning models may not fully resolve.

In conclusion, our experimental design explicitly decouples the contribution of feature engineering from model architecture. While Figure 3 demonstrates that interaction-centric encoding is a prerequisite for any model to achieve predictive power, Figure 4 isolates the architectural advantage. By equipping all baseline models with this optimal encoding, we show that PAIRNet's better performance is driven by its hybrid CNN-Transformer design, which resolves complex local motifs and global dependencies that standard machine learning models even with the correct input features, cannot fully capture.

Minor comments:

Q1. Fig 1A. Mismatch and indel are not present intuitively. I would suggest a dark dot as mismatch, a "–" indicates deletions, and a "-AT-" as insertions.

Response:

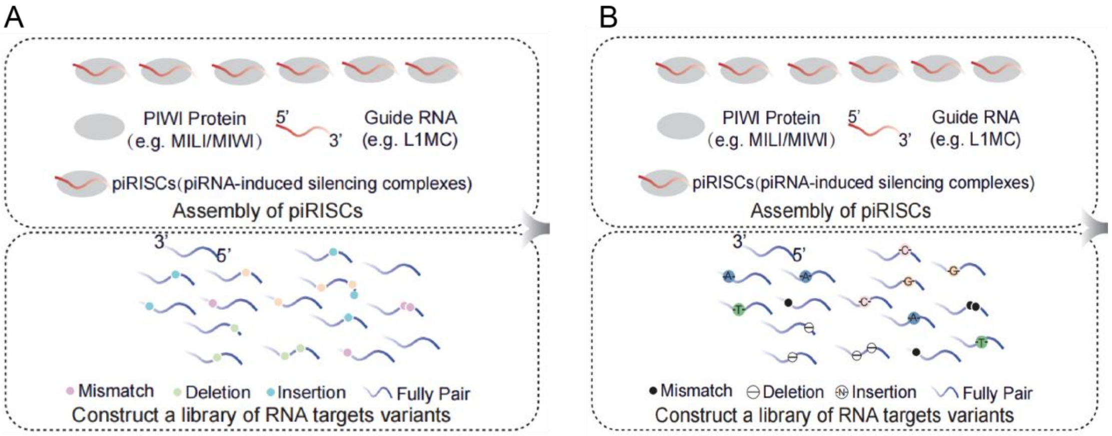

Figure R5. (A) A screen capture for Figure 1A in initial submission, using dots with different color indicate the mismatch, deletion, insertion. (B) Following reviewer's suggestions, black dot, dash with letters and small dashes are used to intuitively symbolize these mutation events.

We thank the reviewer for this suggestion to improve visual clarity. We have updated Figure 1A to adopt the suggested notation: mismatches are now indicated by dark dots, deletions by dashes ("–"), and insertions by explicitly labeled nucleotide sequences (e.g., "-AT-") to make the schematic more intuitive. We have replaced the new plot in revised manuscript.

Q2. line 61. "When revisiting MILI/MIWI data in paper" should be "When revisiting MILI/MIWI data in Gainetdinov et al.".

Response:

We accept this correction. We have revised the text on line 61 to cite the authors explicitly as "*Gainetdinov et al*." instead of the generic "in paper".

Q3. line 62-64. It would be better to put Fig S1–4 as different panels, as they support the same conclusion.

Response:

We agree that merging these figures improves the flow of the supplementary material. We have reorganized S1–S4 Figs into a single multi-panel figure (now S1 Fig, Panels A–D) to collectively illustrate the cleavage rate trends and guide-specific variations.

Corresponding Manuscript Revision words revised (Introduction Line # 61**)**

When revisiting MILI/MIWI data in *Gainetdinov et al*, although we successfully reproduced the change in pre-steady-state cleavage rate for single (S1 FigA) or double consecutive mismatches (S1 FigB) for contiguous g2-g21 positions, we still observed variations between different guide RNA both for MILI (S1 FigC) and MIWI (S1 FigD).

Q4. Fig S1 is clipped.

Response:

We apologize for this rendering error. We have corrected the figure file to ensure S1 Fig is fully visible and properly formatted.

Q5. line 64. "we still observed variations 63 between different guide RNA both for MILI (S3 Fig) and MIWI (S4 Fig)." Do you mean Fig S1–2?

Response:

We originally cited S3 and S4 here because they specifically break down the data by individual guide RNA (showing distinct distributions for L1MC, Kctd7, etc.), whereas S1 and S2 showed the aggregated trends. However, to avoid confusion and aligned with the revision in Q3, these are now presented as panels within the unified S1 Fig, and the text has been updated to reference the specific panels (e.g., S1C and S1D Fig) that display these guide-specific variations.

Corresponding Manuscript Revision words revised (Introduction Line # 61**)**

When revisiting MILI/MIWI data in *Gainetdinov et al*, although we successfully reproduced the change in pre-steady-state cleavage rate for single (S1 Fig. A) or double consecutive mismatches (S1 Fig. B) for contiguous g2-g21 positions, we still observed variations between different guide RNA both for MILI (S1 Fig. C) and MIWI (S1 Fig. D).

Q6. line66. Cite Figures.

Response:

We have added the appropriate figure citations to line 66 as requested.

Corresponding Manuscript Revision words revised (Introduction Line # 66**)**

Specially, We observed a clear deviation in cleavage performance when loading Kctd7 or piRNA2 compared to L1MC and piRNA1 at the g10 (S1 FigD).

Q7. Fig. 1C. What is the difference between concatenation and CNN-based embeddings?

Response:

Concatenation-based encoding flattens the one-hot encoded sequence matrix (26×4) into a single long vector (1×104), treating each nucleotide position as an independent feature input for the model. CNN-based embedding retains the 2D structure of the sequence and applies a 1D-convolutional layer to extract local sequence motifs (e.g., *k*-mers) before passing the learned features to the classifier. The "CNN" bar in Figure 3 refers to this specific embedding strategy applied to raw sequences, which failed to capture interaction rules compared to our interaction-centric approach. We have clarified this distinction in the figure legend and methods.

Q8. line95. Typo: "initeraction".

Response:

We have corrected the typo to "interaction".

Q9. Fig 6. "NoPosition" and "Position Embedding" should be clearly indicated in the figure title or legend

Response:

We have revised the title and legend of Figure 6 to explicitly label the panels as "With Position Embedding" and "NoPosition (Ablation)" to clearly distinguish the experimental setups .

Corresponding Manuscript Revision words revised (Figure 6 Caption Line # 456**):**

Feature importance comparison between the full PAIRNet model and the NoPosition ablation variant. Bar plots show the saliency score of each position of guide RNA using mixed model, integrating data from 4 guides of MILI and MIWI (Saliency score, mean ± SD, *n*=10). (A) With Position Embedding: Saliency scores derived from the full PAIRNet model, which includes learnable positional encodings. (B) NoPosition (Ablation): Saliency scores derived from the ablated model where position embeddings were removed, showing a uniform distribution of importance due to the loss of spatial context.

Reviewer #3:

The manuscript describes a new ML approach to predict rates for piRNA-guided cleavage. Small RNA sequence influences AGO/PIWI-catalyzed cleavage rates (PMID: 39025072 and PMID: 37344600). The important goal of this work appears to be finding a unifying rule set for piRNAs of different sequences. It is a useful study, but several major concerns need to be addressed:

Q1. If authors hold out one piRNA (e.g., L1MC) and train the model without it, will the 3-piRNA (piRNA#1, piRNA#2, Kctd7) model predict how the fourth piRNA cleaves? The hold-out experiment should be repeated for each of the four piRNAs.

Response:

We thank the reviewer for emphasizing this critical validation step. We confirm that this "Leave-One-Guide-Out" strategy was the primary method we used to validate PAIRNet's generalization capabilities in the original submission. In our manuscript, we referred to this as testing on "hold-out datasets" (distinct from the "mixed" dataset). As detailed in the Results section and visualized in Figure 4 and Figure 5:

We trained independent models where one specific guide (e.g., L1MC/Dataset 1) was completely excluded from the training set (which consisted of the remaining three

guides). We then tested the model on that unseen guide to assess generalization. This process was repeated for all four guides (Dataset 1: L1MC, Dataset 2: Kctd7, Dataset 3: piRNA#1, and Dataset 4: piRNA#2).

The results shown in Figure 4 (specifically the first four groups of bars for each protein) directly display the performance of these “held-out” models. For instance, PAIRNet achieves a Pearson Correlation Coefficient (PCC) of roughly 0.6–0.7 for the unseen L1MC guide (Dataset 1) in MIWI experiments, demonstrating that the model successfully learns targeting rules from the other three guides and generalizes them to a completely new sequence. We have revised the text in the Materials and Methods section to explicitly use the term "Leave-One-Guide-Out cross-validation" to avoid any ambiguity regarding the “hold-out” terminology.

Corresponding Manuscript Revision red colored words inserted (Materials and Methods Line # 339**):**

For the second stage, models were evaluated via Leave-One-Guide-Out cross-validation. In this setup, one guide RNA (and all its associated targets) was completely excluded from the training set and reserved for testing to assess the model's ability to generalize to unseen guide sequences. Cleavage rates (*k*) were standardized using z-score normalization (mean=0, variance=1) during training. The best epoch was selected by minimizing the average validation MAE across folds, with final models retrained on the full training set and tested on held-out data... (the rest of this paragraph remains untouched)

Q2: Do figures 6 and 7 show data from all models (four datasets and two proteins as well as mixed)? If so, it would be important to highlight the data from the mixed model, as such a model would reflect more general rules

Response:

We thank the reviewer for this important clarification regarding the scope of our interpretability analyses.

Regarding Figure 6 (Saliency Maps): Yes. We confirm that Figure 6A utilizes the Mixed Model, trained on the pooled dataset including all guides across both MIWI and MILI. This analysis integrated a total of 520 data samples (balanced equally between MIWI and MILI, 10 iterations for each position) to calculate gradient-based saliency. By using the mixed model here, Figure 6A successfully summarizes the “general rules” of PIWI targeting, highlighting the universal importance of the catalytic core (g10–g11) irrespective of the specific protein or guide sequence.

Regarding Figure 7 (Counterfactual Analysis): The data presented in this figure are derived specifically from the MILI-trained model. We selected this model to ensure direct compatibility with the alignment template, which is a Cryo-EM structure of the MILI-piRNA-target complex (PDB: 9IJ3). By mapping MILI-specific computational predictions directly onto the corresponding MILI protein structure, we maintain structural consistency and avoid potential artifacts that could arise from

aligning general PIWI predictions to a specific protein conformation.

However, following the reviewer's suggestion to seek general rules, we have now performed the counterfactual analysis using the Mixed Model (integrating both MIWI and MILI data).

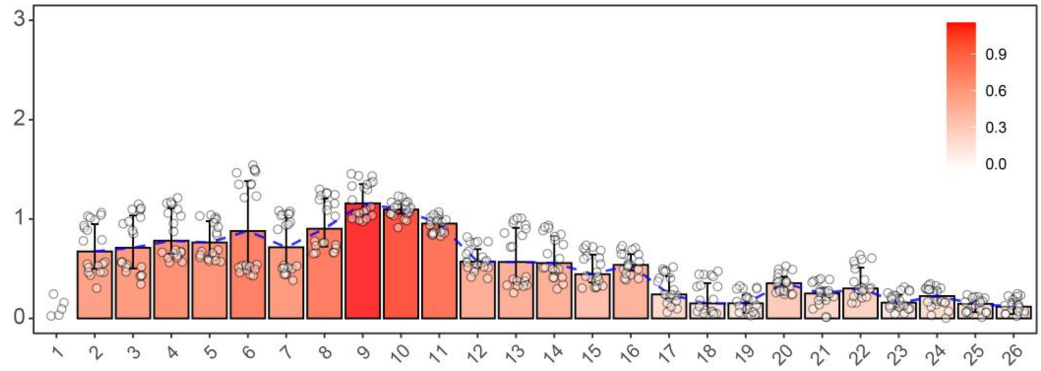

Figure R6. Counterfactual analysis of position importance analyzed for mixed model integrating both MIWI and MILI data.

Figure R6 shows that the mixed model retains the feature importance profile centered on the catalytic core. However, the pooled analysis exhibits higher variance compared to the single-protein models. This variation likely reflects kinetic differences between MIWI and MILI. Although both proteins share the relaxed targeting' mechanism, protein-specific sensitivities to mismatches may manifest as noise when the datasets are combined.

Corresponding Manuscript Revision words revised (Figure 6 Caption Line # 456**):**

Fig 6. Feature importance comparison between the full PAIRNet model and the NoPosition ablation variant. Bar plots show the saliency score of each position of guide RNA using mixed model, integrating data from 4 guides of MILI and MIWI (Saliency score, mean ± SD, *n*=10). (A) With Position Embedding: Saliency scores derived from the full PAIRNet model, which includes learnable positional encodings. (B) NoPosition (Ablation): Saliency scores derived from the ablated model where position embeddings were removed, showing a uniform distribution of importance due to the loss of spatial context.

Corresponding Manuscript Revision words inserted (Figure 7 Caption Line # 489**):**

Fig 7. Alignment of Position Impact by Counterfactual Analysis Result to Structural Insight of piRISC. (A) showed median impact score of each 1-based guide position from g1 to g26 derived from the MILI-specific PAIRNet model; (B) showed three sections (g1, g8 to g14 and 3'end) were captured from Cryo-EM-derived 3D structure with PDB entry 9ij3 (See S3 Fig for Counterfactual Analysis integrating MILI and MIWI).

<u>Q3. Authors could test how their model predicts data from another cleave'n'seq experiment described in PMID: 40912244</u>

Authors' Response:

We thank the reviewer for referring the recent study by *De et al.* (2025) [4]. We agree that evaluating PAIRNet against diverse PIWI homologs is important to define the model's scope. However, a direct quantitative comparison was not possible, because the data are not publicly available. Moreover, the study by *De et al.* (2025) is made on Silkworm, while the data in our manuscript are from Mouse. The two species are very different.

To generate specific predictions, our model requires the guide sequence, the target sequence, and the ground-truth cleavage metric for each pair. While *De et al.* described a library of ~1,024 target RNAs [4], the specific sequences and their corresponding per-target cleavage metrics have not been provided in the Supplementary Information. We contacted the corresponding author (Dr. Ian J. MacRae) to request the processed source data but have not received a response as of this submission (See Figure R7). Consequently, a ground-truth validation could not be performed.

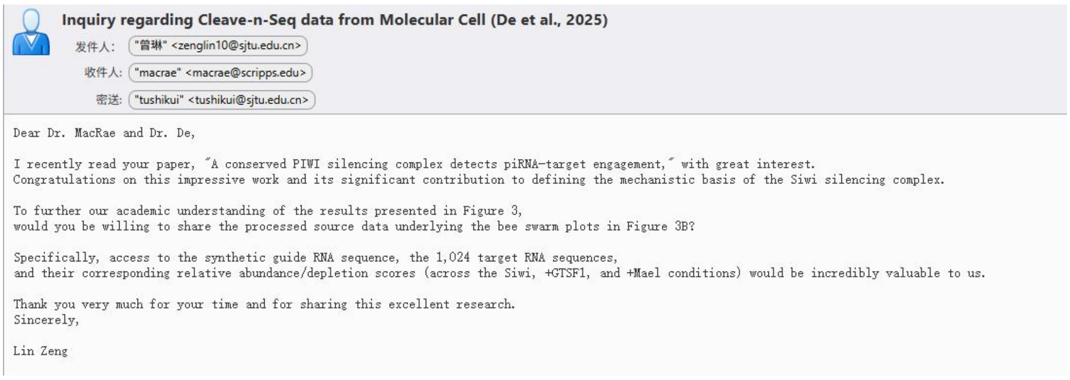
Inquiry regarding Cleave-n-Seq data from Molecular Cell (De et al., 2025)
发件人: "曾琳" <zenglin10@sjtu.edu.cn>
收件人: "macrae" <macrae@scripps.edu>
密送: "tushikui" <tushikui@sjtu.edu.cn>

Dear Dr. MacRae and Dr. De,

I recently read your paper, "A conserved PIWI silencing complex detects piRNA-target engagement," with great interest.
Congratulations on this impressive work and its significant contribution to defining the mechanistic basis of the Siwi silencing complex.

To further our academic understanding of the results presented in Figure 3,
would you be willing to share the processed source data underlying the bee swarm plots in Figure 3B?

Specifically, access to the synthetic guide RNA sequence, the 1,024 target RNA sequences,
and their corresponding relative abundance/depletion scores (across the Siwi, +GTSF1, and +Mael conditions) would be incredibly valuable to us.

Thank you very much for your time and for sharing this excellent research.
Sincerely,

Lin Zeng

Figure R7. Screen capture for emailing to author of paper PMID 40912244. We hope to get source data for us to do integrative test on this CNS-seq data of Siwi-GTSF1-Maelstrom complex but have not received a response since Nov. 22, 2025.

To try our best to address the reviewer's concern, we simulated the *De et al.* experimental design using our Mouse-trained model. The *De et al.* library utilizes a constant, perfectly matched region spanning the seed (g2–g15) and randomizes only the 3' end (g16–g25). When applied to this sequence space, PAIRNet predicted high cleavage rates with low variance across the libraries, which indicates an inability to distinguish targets based solely on 3' status. The result is expected and it validates that the model has correctly learned the Mouse PIWI mechanism rather than the Silkworm mechanism.

Biological Incompatibility (Mouse vs. Silkworm): The inability of the Mouse-trained model to predict Silkworm data reflects the distinct activation mechanisms established in the two papers. *De et al.*[4] established that Siwi exists in a 'locked', catalytically inactive state where the catalytic residue (E708) is sequestered

from the active site, extended 3' pairing is strictly required to structurally 'unlock' the protein and recruit GTSF1/Maelstrom to position E708 for catalysis[4]; conversely, the *Gainetdinov et al.* data used to train PAIRNet establishes that Mouse PIWI proteins operate via a 'relaxed' mechanism. While they utilize GTSF1, they do not require extensive 3' pairing as an obligate binary switch for catalysis; instead, they tolerate mismatches and are driven primarily by seed affinity.

Since PAIRNet learns the Mouse mechanism, where a perfect g2–g15 match is sufficient for high-affinity binding and cleavage, it views the *De et al.* library (which contains perfect g2–g15 matches) as uniformly high-quality targets. The model essentially confirms that it has not learned the Silkworm-specific 3' activation switch, which is absent in mammalian biology. We have added a discussion of these species-specific mechanistic differences to the manuscript to clarify the model's biological scope.

Corresponding Manuscript Revision words inserted (Discussion Line # 564**):**

Furthermore, PAIRNet is trained on mammalian PIWI data, which reflects a 'relaxed' targeting mechanism. Consequently, the current model may not fully generalize to other systems, such as the silkworm Siwi-RE, where a 'locked' conformation requires extensive 3' pairing to structurally activate the complex....Future work should expand to in vivo cleavage assays, additional PIWI homologs (e.g., HIWI, HILI), and other species-specific data.

Reference

[1] Wang, P.Y. and D.P. Bartel, *The guide-RNA sequence dictates the slicing kinetics and conformational dynamics of the Argonaute silencing complex.* Mol Cell, 2024. **84**(15): p. 2918-2934 e11.

[2] Becker, W.R., et al., *High-Throughput Analysis Reveals Rules for Target RNA Binding and Cleavage by AGO2.* Mol Cell, 2019. **75**(4): p. 741-755 e11.

[3] Gainetdinov, I., et al., *Relaxed targeting rules help PIWI proteins silence transposons.* Nature, 2023. **619**(7969): p. 394-402.

[4] De, D., et al., *A conserved PIWI silencing complex detects piRNA-target engagement.* Mol Cell, 2025. **85**(17): p. 3275-3287 e7.
